# The Influence of the Shielding-Gas Flow Rate on the Mechanical Properties of TIG-Welded Butt Joints of Commercially Pure Grade 1 Titanium

**DOI:** 10.3390/ma17051217

**Published:** 2024-03-06

**Authors:** Krzysztof Szwajka, Joanna Zielińska-Szwajka, Tomasz Trzepieciński

**Affiliations:** 1 Department of Integrated Design and Tribology Systems, Faculty of Mechanics and Technology, Rzeszow University of Technology, ul. Kwiatkowskiego 4, 37-450 Stalowa Wola, Poland; kszwajka@prz.edu.pl; 2 Department of Component Manufacturing and Production Organization, Faculty of Mechanics and Technology, Rzeszow University of Technology, ul. Kwiatkowskiego 4, 37-450 Stalowa Wola, Poland; j.zielinska@prz.edu.pl; 3 Department of Manufacturing Processes and Production Engineering, Rzeszow University of Technology, al. Powstańców Warszawy 8, 35-959 Rzeszów, Poland

**Keywords:** hardness, mechanical properties, microstructure, titanium, welded joints, welding

## Abstract

This article proposes as a novelty the differentiation of shielding-gas flow rates from both sides of the tungsten inert gas (TIG)-welded butt joints of commercially pure (CP) grade 1 titanium tubes. Such an approach is aimed at economically reducing the amount of protective gas used in TIG closed butt welding. The effect of the shielding-gas flow rate on the properties of CP grade 1 titanium butt-welded joints made using the tungsten inert gas (TIG)-welding method. Butt-welded joints were made for different values of the shielding-gas flow from the side of the root of the weld. Argon 5.0 was used as the shielding gas in the welding process. As part of the research, the welded joints obtained were analysed using optical and scanning electron microscopy. The microstructural characteristics of the joints were examined using an optical microscope, and the mechanical properties were determined using hardness and tensile tests. It was observed that as the flow of the shielding gas decreases, the hardness of the weld material increases and its brittleness also increases. A similar trend related to the amount of gas flow was also noticeable for the tensile strength of the joints. The increase in the hardness of the weld and the heat-affected zone compared to the base metal is mainly related to the increase in the amount of acicular structure (α′ phase). The optimal gas flow rates from the side of the root of weld were found at the values of 12 dm^3^/min.

## 1. Introduction

The high strength, low weight and exceptional corrosion resistance of titanium and titanium alloys have led to a wide and diverse range of applications in the aerospace industry [1], chemical engineering [2], the automotive industry [3], marine applications [2], biomedical applications [4] and sports equipment [5]. Titanium and its alloys can be classified into five groups according to their microstructure [6]: single-phase α alloys, two-phase α+β alloys, single-phase β alloys, pseudo α alloys (consisting of the α phase and a small content of elements stabilising the β phase—that is, Cr, Mo, V, Cu, Ru) and pseudo β alloys containing quite a large amount of elements stabilising the β phase (i.e., Ga, Al, Ge), which causes a shift of the β → α phase transformation to lower temperatures. Pure titanium has an α structure at room temperature [7].

Titanium is a ductile alloy that can be formed by sheet metal methods [8] or other plastic working techniques [9]. Due to the hexagonal close-packed crystal structure, titanium alloys exhibit a low ductility and formability at room temperature [10]. However, at elevated temperatures, the titanium and Ti-based alloys can be successfully formed [11]. Some titanium components require joining. Welding is a quick and easy method of joining metals. Titanium and its alloys are joined using various methods: tungsten inert gas (TIG) welding [12,13], metal inert gas (MIG) welding [14], plasma arc welding (PAW) [15], laser beam (LB) welding [16], flux-cored arc (FCA) welding [17] and electron beam (EB) welding [18]. When selecting a welding method, the specific properties of titanium, which determine various difficulties affecting the weldability of titanium, should be considered [19,20]:

- Very high chemical activity at elevated temperatures with carbon, nitrogen, oxygen and hydrogen makes it necessary to use a protective atmosphere;
- The high melting point of titanium requires the use of concentrated heat sources;
- The tendency to grain growth when heated to high temperatures, which is characteristic of the titanium-welding process in terms of β phase stability (>880 °C);
- Prolonged exposure to high temperature causes grain growth.

TIG welding allows an extremely clean and high-quality weld to be obtained [21]. No slag is produced, eliminating the risk of weld contamination, and therefore, the finished weld requires no cleaning. The TIG method is most often used for welding stainless steels and other high-alloy steels and materials allied with aluminium, copper, titanium, nickel, magnesium and their alloys [22,23]. This method is generally used for welding pipes and pipelines as well as thin sheets.

Research on the joining technology and operational properties of titanium and its alloys has been conducted for many years. Rogalski et al. [19] developed the procedure of TIG welding of a shell and a tube heat exchanger made of Grade 2 titanium. The authors concluded that the key to obtaining good-quality joints is the preparation of the surface and the application of a special cleaning regime to the welding area. Experiments on TIG welding of Grade 2 titanium sheets conducted by Raja et al. [13] revealed that the mechanical properties of butt joints are greatly affected by the temperature used during the welding process. Szymlek [20] analysed the hardness and mechanical properties of Grade 2 titanium butt joints fabricated using TIG welding. He found that the increase in the hardness of the weld and the heat-affected zone (HAZ) compared to the base metal (BM) is mainly related to the increase in the amount of acicular structure (α′ phase). Subramaniyan et al. [24] employed the TIG-welding process to fabricate the butt joint of Ti-6Al-4V titanium alloy sheets. They found that the high angle–grain boundary proportion of the fusion zone (FZ) increases with the volume of applied heat. The results of TIG welding of the Ti-6Al-4V titanium alloy showed that the martensitic microstructure in the weld resulted in an increased hardness within the FZ and heat-affected zone [25]. Niagaj [26] investigated the effect of activating flux and selected fluorides on activated TIG (ATIG) welding of Grade 2 titanium. The application of BC-Ti activating flux caused an increase in the penetration depth compared to conventional TIG welding. Gurevich et al. [27] found that the increase in the penetration depth in TIG welding could be attributed to the application of the salts of alkali metals. Patil et al. [28] studied the effect of gas flow rate, weld gap and weld current on the mechanical properties of CP Grade 2 titanium TIG-welded joints. A significant grain coarsening was found in the FZ consisting of an α phase bounded by a β phase. Choi and Choi [29] investigated the effect of weld passes and the flow rate of the shielding gas on the hardness of Grade 2 pure titanium material TIG-welded. The hardness value at the HAZ was higher than that for the weld zone. Bendikiene et al. [30] compared pulsed TIG welding in short interrupted intervals and the conventional TIG-welding process of CP titanium plates. Radiographic assessment of the welds fabricated using pulsed TIG welding revealed elongated cavities, gas pores, transverse cracks and porosity, which did not meet quality requirements. Otani [31] proposed a back-shielding device at the back side of torch to protect the weld. Ren et al. [32] used TIG welding to create the joint near α-Ti-based TA15 alloy. It was found that the martensite α′ phase formed in the HAZ and FZ can increase the hardness of the weld. Karpagaraj et al. [33] investigated the mechanical properties of TIG-welded CP titanium. The results revealed that the hardness value in the FZ is bigger than for the BM. Wu et al. [34] numerically analysed the temperature distribution and energy propagation during tandem TIG welding of titanium tubes. The calculated arc efficiency in the analysed TIG-welding process was 79.8%. It was also found that due to the Marangoni stress, the molten metal flowed backwards on the top weld pool surface.

Titanium and its alloys are materials widely used in the construction of aircrafts, space rockets, jet engines and in the petroleum industry. Tungsten inert gas (TIG)-welding technology is most often used to join sheets from titanium and its alloys. At high temperatures, titanium and its alloys react very easily with elements found in the air, such as oxygen, nitrogen and hydrogen. Moreover, titanium is chemically reactive with atmospheric gases above 120 °C, which requires special attention during welding. In order to prevent contamination to the titanium, parts heated to high temperatures should be covered with inactive protective gases [35]. However, it must be remembered that, in such a case, there is also a risk of contamination of the weld by impurities in the gas or air entering the welding zone due to the instability of the gas shield. When selecting a welding method, the specific properties of titanium, which determine various difficulties affecting the weldability of titanium, should be considered [20,21]. The tendency to grain growth when heated to high temperatures is characteristic of the titanium-welding process in terms of β phase stability (>880 °C); prolonged exposure to high temperature causes grain growth.

The effect of current, welding speed, voltage and shielding-gas flow rate on the mechanical properties of the TIG-welded joint constitute the main research direction. TIG welding of titanium sheets is carried out in chambers, ensuring the same flow rate of the shielding gas from the side of the face of the weld and from the side of the root of the weld.

This article proposes as a novelty the differentiation of shielding-gas flow rate from both sides of the weld. Such an approach is aimed at reducing the amount of protective gas used in TIG closed butt welding of commercially pure (CP) Grade 1 titanium joints. This approach allows for an economical reduction in the amount of gas necessary for TIG welding and is consistent with sustainable manufacturing. The effect of different amounts of Argon 5.0 shielding-gas flows used on the side of the root of the weld on the microstructure, grain size and mechanical properties (microhardness and ultimate tensile strength) of the joints was tested. The view of the welds on the face of the weld and on the root of the weld was thoroughly characterised.

## 2. Test Material and Methods

### 2.1. Material

**Figure 1:**
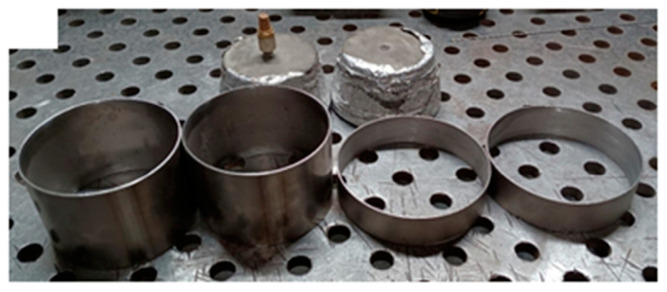
The shape of the CP (Grade 1) titanium specimens.

In the tests, a tube made of commercially pure Grade 1 titanium with an outer diameter of 102 mm and a wall thickness of 1 mm was used as the welded material (Figure 1). After annealing, titanium may have an acicular or equiaxed morphology. The chemical composition of the welded material (Table 1) was determined by X-ray fluorescence (XRF) analysis on an Epsilon analyser (Malvern Panalytical, Malvern, UK).

The XRF method depends on fundamental principles that are common to XRD and X-ray spectroscopy (SEM-EDS) [36]. The analysis of the chemical composition of materials by XRF is made possible by the behaviour of atoms when they interact with X-radiation. If a sample is illuminated by an intense X-ray beam, some of the energy is scattered, but some is also absorbed within the sample in a manner that depends on its chemistry. Primary X-rays are generated by the source and directed to the sample surface. When the beam hits atoms in the sample, they react to generate secondary X-rays, which are collected and processed by a detector. The energy of the emitted X-ray radiation is characteristic for each element [37]. X-rays emitted by atoms in the sample are collected by a detector and processed in an analyser to generate a spectrum showing peaks in X-ray intensity as a function of their energy. The intensity of a peak indicates its amount in the sample. The analyser then uses this information to calculate the elemental composition of the sample. A detailed theoretical description of how the (XRF) analyser works can be found in [36].

Before starting the main tests, the basic mechanical properties of the test material were measured using a uniaxial tensile test performed at the Zwick/Roell Z100 universal testing machine (ZwickRoell, Ulm, Germany) according to the EN ISO 6892-1:2019 [38] international standard. The Vickers’ hardness of the test material was measured using a Qness 60M hardness tester (QATM—Materialography & Hardness Testing, Mammelzen, Germany) in accordance with the ISO 6507-1:2023 [39] standard. The tests were repeated three times and the average values of selected mechanical parameters (Table 2) were determined. The energy dispersive X-ray (EDX) analysis of the elements constituting the material was carried out using a Tescan^®^ MIRA3 scanning electron microscope (SEM) (Tescan, Brno, Czech Republic). 

### 2.2. Welding Procedure

**Figure 2:**
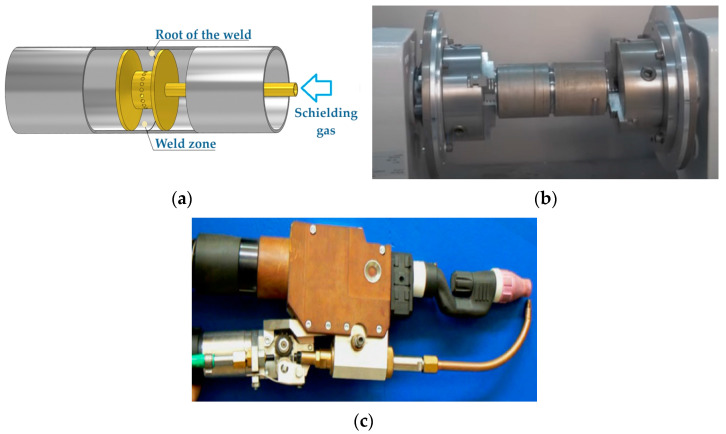
(**a**) The supply of the shielding gas from the side of the root of weld, (**b**) the welding positioner and (**c**) the TIG torch.

The welding process was carried out on a robotic station, a TruLaser Robot 5020 (TRUMPF, Ditzingen, Germany) equipped with a TIG torch (Figure 2c). The MasterTig AC/DC welding device (KEMPPI, Lahti, Finland) was used as the power source for the TIG welding. A non-consumable WL20 2.4 × 175 tungsten electrode with the addition of 2% lanthanum oxide was used for welding. The gas nozzle used for the tests is a lens nozzle with a diameter of 20 mm. The shielding gas used was Argon 5.0 with a purity of 99.999%. Ti-0.15O2 240 ERTi-1 wire with a diameter of 1 mm was used as the filler metal.

Both connected tubes were mounted in the welding positioner (Figure 2b). During the welding process at the robotised station, the tubes rotated around a common axis with an adjustable rotational speed. The welding process was carried out by a robot arm with a welding handle installed. The initial joint welded using the TIG method was made using a current of I = 65 A, a welding speed of 12 cm/min, a voltage U = 12 V and a shielding-gas flow rate (Argon 5.0) on the face of the weld of 15 dm^3^/min and on the side of weld root of 18 dm^3^/min. The face surface of the obtained welded joint (for the above welding parameters) was blue and dark brown in colour, indicating oxidation of the weld as a result of insufficient protection against atmospheric air. The root of the weld was properly protected, as evidenced by the silver colour of the weld. Based on visual tests, it was found that the welded joint had a very wide weld (the width of the face of the weld and the width of the root of the weld of 8 mm) and a wide HAZ. The total width of the weld including the HAZ was 12 mm. The next joint was welded using a current of I = 50 A, a voltage of U = 12 V, a welding speed of 3 mm/s and a gas flow rate of 18 dm^3^/min from the face of the weld.

To protect the weld against atmospheric air from the side of the root of the weld, the space inside the tube was closed and the shielding gas supplied through a stub pipe (Figure 2a), while a gas nozzle with a diameter of 20 mm was used on the side of the face of the weld. Both the root and the face of the weld were silver in colour after welding. Changing the welding parameters resulted in a reduction in the width of the weld (the width of the face of the weld was 6 mm and the width of the root of the weld was 3 mm). Using the above-mentioned parameters, welded joints were made, marked with the following names from TIG_1 to TIG_5 (Table 3). The parameter that changed during the welding process was the flow of the shielding gas from the side of the root of the weld. Three repetitions were performed for each shielding-gas flow.

An important parameter in the titanium-welding process is linear energy. This is a measure of the amount of heat needed to make a weld. The amount of heat introduced into the material affects many features of the welded joint including the following:

- The amount of welding stresses and deformations;
- The maximum cooling rate in the HAZ;
- The size of the heat-affected zone;
- The volume of the melted material;
- The impact strength of the joint;
- The maximum hardness.

In the tests carried out, the amount of heat introduced was Q = 0.12 kJ/mm.

### 2.3. Investigation of Joint Strength

**Figure 3:**
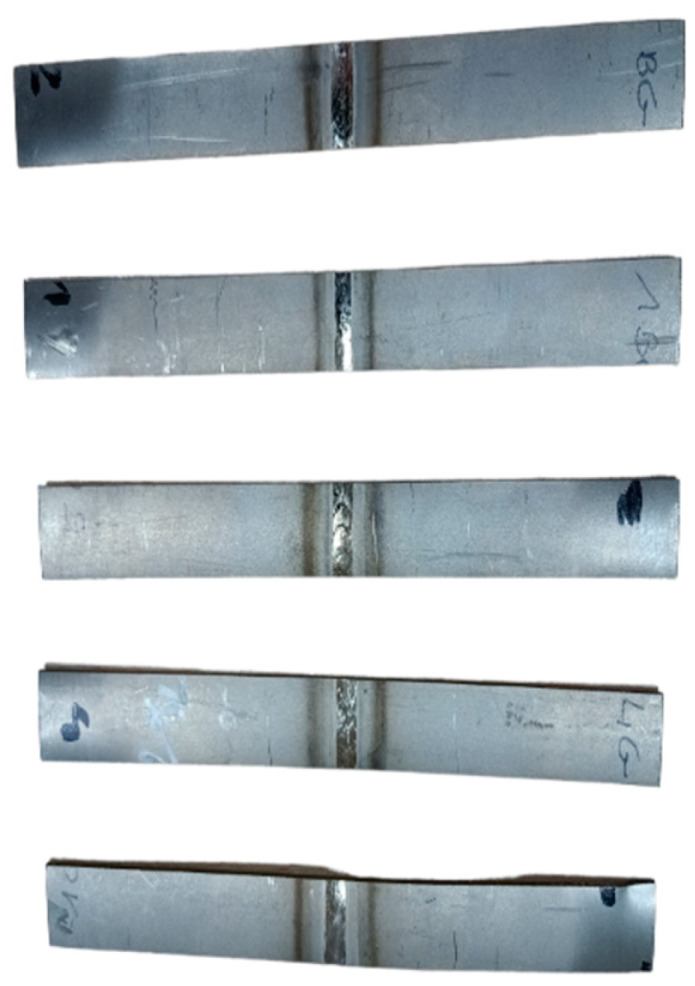
Samples prepared for static tensile testing.

The static tensile test of welded joints was carried out on a Zwick/Roell Z100 testing machine. The static tensile tests were carried out according to the EN ISO 6892-1:2019 [38] standard. Figure 3 shows samples prepared for a static tensile test. The initial length of the samples was L = 150 mm and the width was 15 mm. The longitudinal specimens were extracted from tubes with a 90° angle from the tube weld [40]. The specimens were cut using electrical discharge machining. Then, the rounded specimens were subjected to a uniaxial tensile test [41].

SEM observations were performed on a Tescan^®^ MIRA3 SEM with an EDS attachment. Measurements of the hardness (HV1) distribution of the welded joint in three characteristic areas (base metal, HAZ and weld) were made using the Vickers Qness 60M hardness tester in accordance with the ISO 6507-1:2023 [39] standard. A load of 9.807 N (Vickers hardness test with low force) and a test time of 10 s was used.

The microstructure was assessed using an optical microscope (OM) and an SEM on a cross-section of welded joints. Metallographic observations were carried out under an Olympus BX51M OM (Olympus Life Science, Tokyo, Japan) coupled with a digital camera and a computer with Olympus Stream Essentials software Version 2.4 with magnifications ranging from 50× to 1000×.

**Figure 4:**
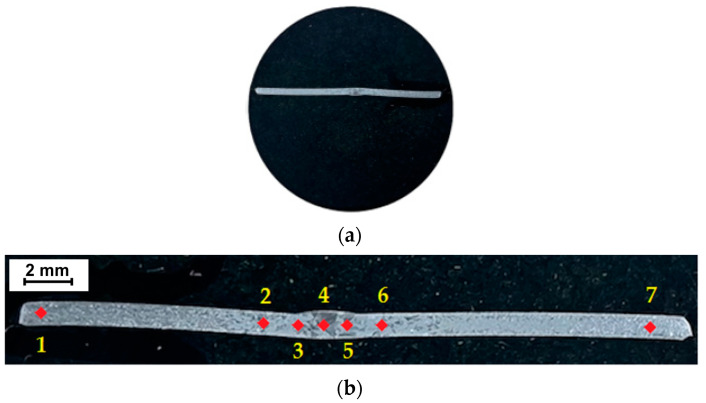
(**a**) The positioned specimen and (**b**) the hardness measurement locations (1–7).

Both for the analysis of the chemical composition and for subsequent analyses in terms of microstructural analysis, the methodology for preparing samples for testing was identical. The test samples were cut from welded tubes on a Mitsubishi Electric MV1200S electro-discharge cutting machine (Mitsubishi Electric, Tokyo, Japan), thus avoiding thermal impact during cutting. A strip perpendicular to the joint length was cut from each joint and embedded in epoxy resin (Figure 4a) to characterise the microstructure cross-section of the joint. Samples intended for metallographic testing were mechanically wet-polished with 220~2200 grit paper. Finally, diamond paste (grain size 1 μm and 3 μm) was used to polish the sample surfaces. Kroll’s reagent was used to etch the samples with the following chemical composition: 2 mL HF, 6 mL HNO_3_, 100 mL H_2_O. The samples were etched at room temperature for approximately 40 s to enable subsequent observation of the microstructure. The hardness was measured at seven points as shown in Figure 4b.

**Figure 5:**
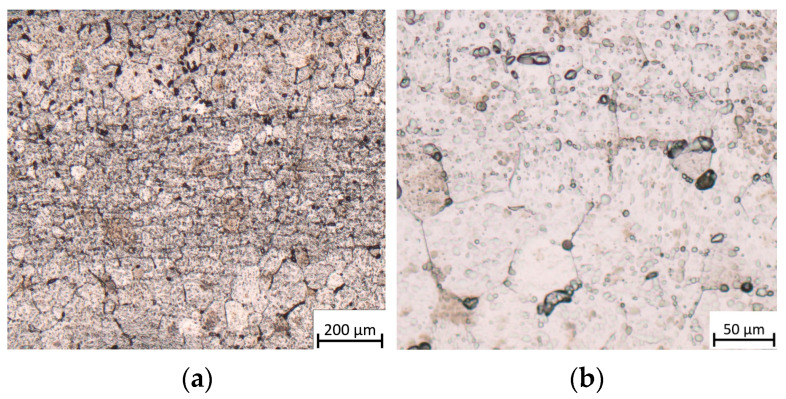
(**a**,**b**) Optical microstructures of the test material.

The microstructure of the base material consisted of equiaxed α grains with an average grain size of 68 µm and a small but noticeable amount of β phase around the grain boundaries. The presence of β phase in CP titanium may result from and be related to the cold-rolling process during the production process. In both photos (Figure 5a,b), the bright phase corresponds to the α phase and the dark phase corresponds to the β phase. Based on image analysis, it was determined that CP (Grade 1) contains approximately 4% of the β phase.

Hardness measurements of the welded joints were carried out in accordance with the ISO 9015-1:2001 [42] standard. The minimum measured hardness of the material on the HV1 scale was 125, and the maximum value was 132.

## 3. Results and Discussion

### 3.1. Microstructure of BM

**Figure 6:**
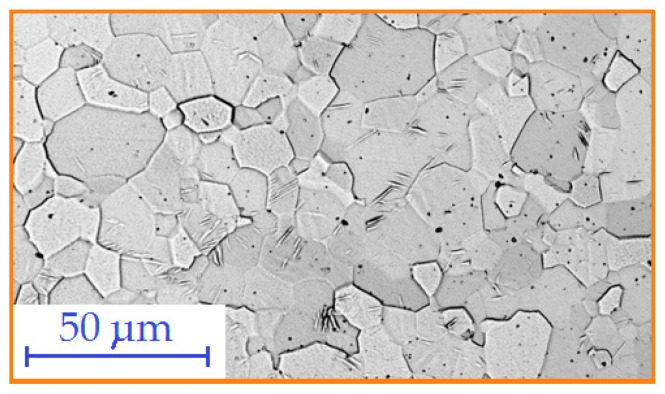
Microstructure of the base metal.

The microstructure of CP Grade 1 titanium consists of equiaxed α phase with a small volume fraction of the β phase (typically less than 2%). Variable size of the average grain size in Grade 1 titanium was observed. The microstructure shown in Figure 6 shows the different grain sizes of the welded material. The average grain size determined for the tested microstructure was 27.3 ± 4.8 µm.

### 3.2. Hardness of the Welded Joint

**Figure 7:**
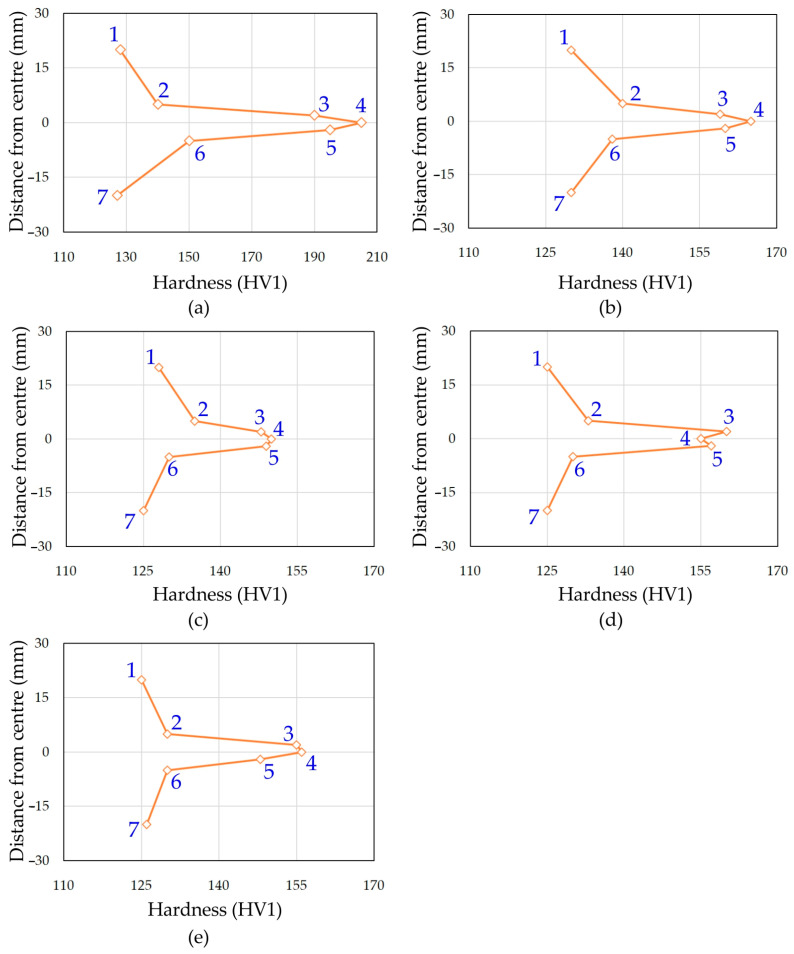
The results of the microhardness measurements of the welded joints for (**a**) TIG_1, (**b**) TIG_2, (**c**) TIG_3, (**d**) TIG_4 and (**e**) TIG_5.

The hardness profiles obtained using the Vickers method for welded joints made in the conducted tests are shown in Figure 7. Hardness measurements of the welded joints were carried out in accordance with the ISO 9015-1:2001 [42] standard. As a result of the hardness measurement tests, it was found that the average hardness values of the base metal are 125 ± 7 HV1. Hardness measurements were made at 2 mm intervals for measurements no. 2, 3, 4, 5 and 6 (Figure 4b). Measurements no. 1 and 7 were made in the welded material. Based on the hardness measurement results obtained, clear differences were observed depending on the location of the measurement. The highest hardness in joints welded using the TIG method was measured in the weld zone. The hardness of the weld material reached a value of 201 HV1 for TIG_1 weld. The relatively high hardness of the weld in this joint is caused by the presence of a finely acicular α′ phase. In welded joints, the HAZ is characterised by lower hardness (approximately 150 HV1) than the weld because it contains a smaller amount of the acicular structure (α′ phase).

Microhardness profiles for welded joints show different average hardness of the joints obtained depending on the amount of shielding gas. In all welded joints, the average hardness values were clearly correlated with the amount of shielding gas. With an increase in the amount of shielding gas, a decrease in the hardness value of the welded joint was observed both in the HAZ and in the weld. In the Grade 1 welded joints, the difference in average hardness between the weld, the HAZ and the base metal can be explained by the difference in microstructure in these areas.

### 3.3. Static Tensile Test

**Figure 8:**
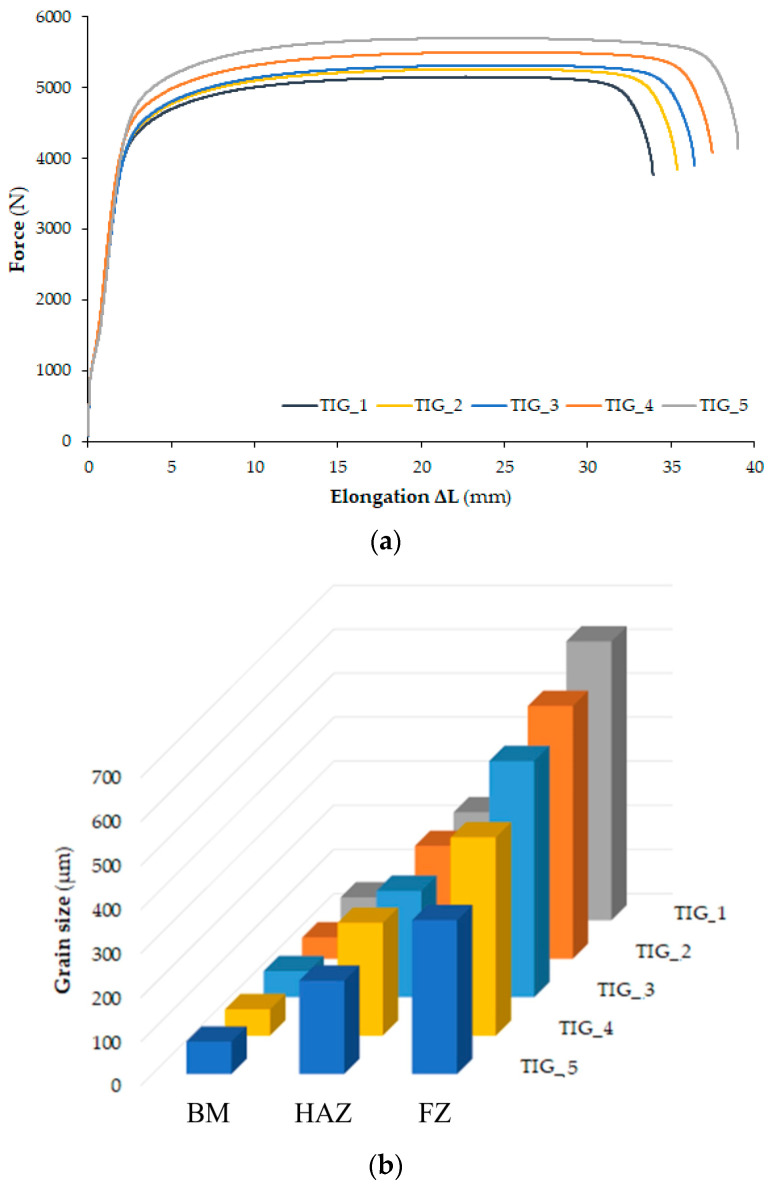
(**a**) Tensile curves and (**b**) and the average grain size in the welded joint.

The cumulative results of ultimate tensile strength (R_m_) obtained with changing values of shielding-gas flow from the side of the root of the weld are presented in Table 4. Additionally, Figure 8a shows the tensile curves for the tested samples.

All experiments were repeated three times. Joints welded using TIG_1 and TIG_2 parameters cracked in the weld zone, which is not the desired (correct) effect due to the strength of the joint. The reason seems to be the presence of an acicular structure (α′ phase) and very large grains of the α phase (Figure 8b). In the remaining tests, TIG_3, TIG_4 and TIG_5, the samples cracked in the base metal.

**Figure 9:**
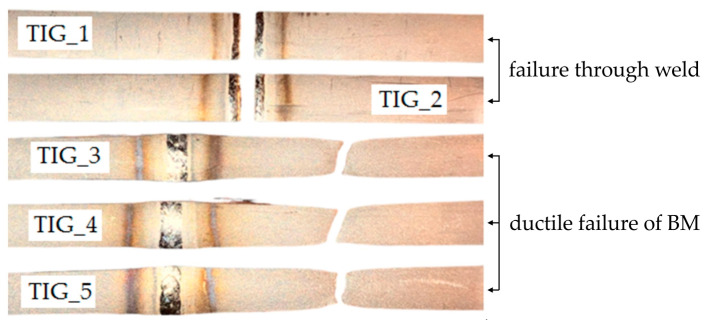
View of samples after the static tensile test.

Figure 8a shows the influence of the shielding-gas flow on the mechanical properties of all the welded joints. In TIG-welded joints, the average tensile strength was between 420 and 481 MPa, and the elongation obtained at the break of the sample was from 33 mm (minimum) to 40 mm (maximum). The mechanical properties depended on the weakest part of the joints, depending on the microstructure of the welded joint. In the joints welded with TIG_1 and TIG_2 parameters, the presence of martensite and porosity in the weld significantly weakened the weld. Therefore, instead of propagating through the relatively weaker HAZ, the crack occurred at the weld zone. Typical fracture surfaces in cup-shaped and cone-shaped configurations are shown in Figure 9. The presence of significant sample narrowing in all three types of joints, TIG_3, TIG_4 and TIG_5, preceded by sample cracking, indicating ductile crack growth behaviour.

### 3.4. Weld Observation

The level of air pollution in the weld can be determined based on the colour of the weld. When maintaining a high heating temperature, the colours change over time in the following order (from low temperature to high): silver, gold, purple, blue, light blue, grey, white, yellow-white. If the colour of the surface is light blue, grey or white, this indicates that the weld metal has become brittle. According to the Japanese Judgement Standard for Technical Certification WES 8104, welds with light blue, grey, white and yellow-white colours are unacceptable.

If the gas shielding is insufficient, the weld metal becomes brittle; so, even if the colour of the weld surface is good, it can cause problems with the load capacity of the joint. The biggest problem when welding titanium and titanium alloys is the occurrence of bubbles and contamination embrittlement caused by air. Small bubbles are easily formed when welding titanium [35]. Therefore, the most important thing is to prevent their formation. The causes of bubbles are inclusions of unpurified gases and impurities located in the additional material, and therefore, to prevent bubbles, welding parameters should be appropriately selected and the welding surface should be cleaned. When welding tubes, contamination and gas leakage inside the tube cannot be allowed.

**Figure 10:**
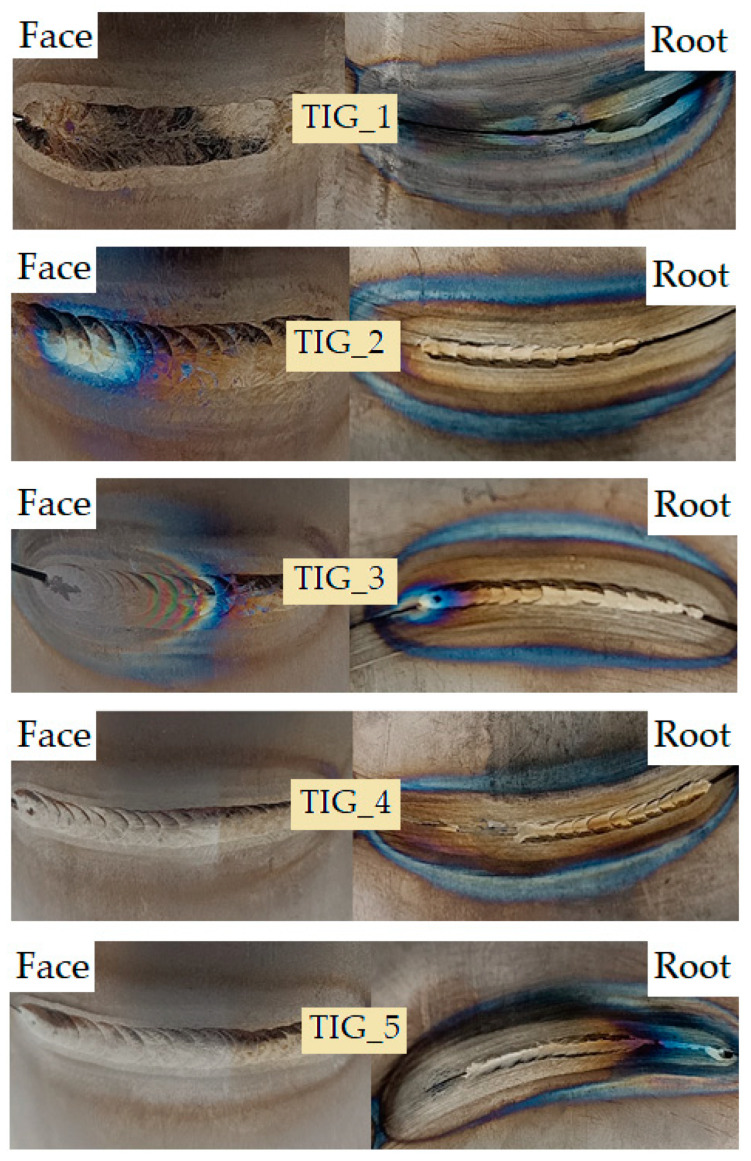
Photographs of welded joints made from the face of the weld and the root of the weld.

Before cutting into the samples, the welded joints (Figure 10) were subjected to visual tests to determine the initial correctness of the joints. The colour of the weld, discoloration, occurrence of fusion and welding imperfections were assessed.

Joint marked TIG_1: The face of the weld is slightly golden. The HAZ is slightly tinted gold. The edge of the joint exposed to the gas shield field is coloured brown. The joint zone directly exposed to the gas shield is coloured in shades of brown with numerous discolorations. The joint from the side of the root of the weld is coloured blue-violet-red-brown. At the melting zone, the cut-off line of the heat-affected zone is clearly outlined. Incomplete melting occurs in one area.

Joint marked TIG_2: The face of the weld is discoloured, from gold through purple to blue. The edge of the gas shield’s impact field is blue-brown. However, the field of direct impact of the gas shield is slightly brown. A short cooling time in the gas shield resulted in discoloration. The root of the weld is silver-gold. The HAZ is coloured gold. The edge of the gas shield influence field from the side of the root of the weld is blue and the area of direct gas shield influence is blue-brown. Root penetration occurs along most of the length of the weld. However, the beginning of the weld does not show complete penetration.

Joint marked TIG_3: The weld is discoloured, the beginning of the weld is silver, then blue-violet. In the arc extinction zone, the weld turned green-pink-grey. The gas flow after the arc was extinguished caused the absorption of oxygen and nitrogen and the formation of a matte finish. The edge of impact of the gas shield field on the side of the root of the weld is brown. The root of the weld is silver-gold in colour. The arc extinction area on the side of the root of the weld is discoloured—the gas shield inside the tube prevented oxidation of this area. The HAZ on the side of the root of the weld is silver-gold in colour. The edge of the gas shield influence zone on the side of the root of the weld is blue, while the zone of direct influence of the gas shield is light brown. Penetration occurs along most of the weld length. However, incomplete penetration occurs at the beginning of the weld. This is due to too little energy being introduced when welding starts.

Joint marked TIG_4: On the side of the face of the weld, the weld is mostly free of discoloration and is silver in colour. The beginning of the weld is coloured gold. This is caused by the disappearance of the gas shield from the burner nozzle in this area. On the side of the face of the weld, the colours of the HAZ and base material are the same. The edge and the area of direct exposure to the gas shield on the side of the face of the weld are coloured light brown. The colour of the weld and the HAZ on the side of the root of the weld is golden. The edge of the area of gas shield influence on the side of the root of the weld is blue. However, the area of direct impact of the gas shield on the side of the root of the weld is coloured brown. Incomplete penetration and partial melting of the root of the weld at the location where welding started was observed. This was caused by too little energy being supplied to the material or too high a welding speed.

Joint marked TIG_5: On the side of the face of the weld, the weld is mostly silver, without discoloration. The beginning of the weld is coloured gold due to the disappearance of the gas shield from the burner nozzle in this area. The HAZ on the face of the weld is consistent with the colour of the base material. The edge of the zone of the gas shield impact is slightly brown, and the area of direct influence of the gas shield does not show any discoloration. The root of the weld is silver-gold-purple-blue. The HAZ on the side of the root of the weld exhibit the colour of the base material. Near the end of the weld, the HAZ is coloured similarly to the root of the weld. At the beginning of the weld, there is no penetration, which turns into incomplete penetration during welding. This is caused by a too high welding speed or too little energy being supplied to the material.

### 3.5. Optical Microscopy

**Figure 11:**
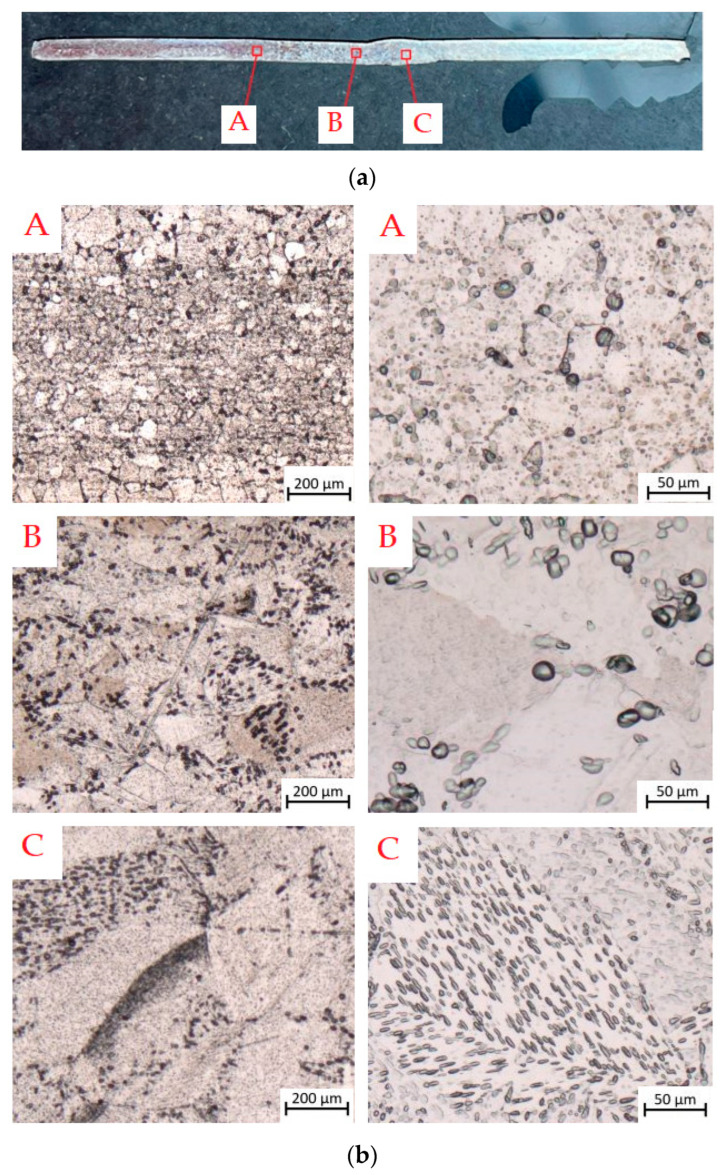
Microstructure of the TIG_3 sample: (**a**) locations of weld assessment and (**b**) views of the microstructure under 5× magnification (**left**) and 20× (**right**).

The joints obtained were subjected to microstructure observations (Figure 11a). Observations were made in three areas: A—base material, B—heat-affected zone and C—weld. The marked places were analysed at magnification of 5× and 20×.

The microstructures of various zones of the welded joint TIG_3 are shown in Figure 11b. This welded joint was made with a shielding-gas flow of 12 dm^3^/min. The microstructure of the heat-affected zone (area B) of the weld is irregular. In the HAZ, where the temperature is high, grain growth can be observed, and most grains have wavy intergranular boundaries. Needle-shaped grains were rarely observed. The grains on the side of the face of the weld have a needle-like morphology. In the centre and at the root of the weld, a weld is formed consisting of columnar grains with an orientation corresponding to the direction of heat dissipation during welding.

**Figure 12:**
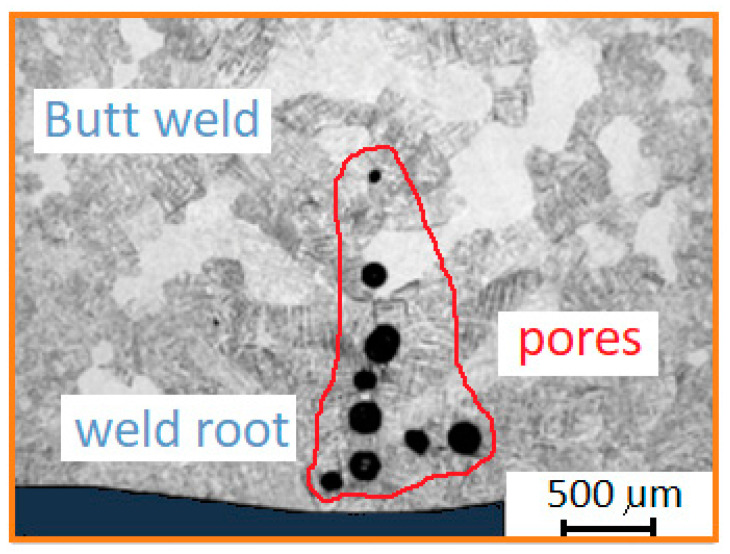
Pores revealed in the TIG_1 weld.

Grain growth occurs in the direction perpendicular to the fusion boundary. A maximum grain size exists in the weld. The temperature in HAZ increases as the distance from the weld pool decreases. The grains in the HAZ microstructure have a polyhedral shape. The apparently larger grains in the HAZ are related to the low cooling rate. There are also fine needle structures in the weld metal. Figure 11b (area C) shows the weld metal. The microstructure shows a fine acicular martensitic structure in the weld metal and coarse grains under the influence of heat. The acicular microstructure was formed as a result of rapid cooling of the β phase. Slower cooling resulted in coarser grains. The largest pore diameter was 150 µm. Due to the lack of full protection of the root of the weld, the weld metal was contaminated by elements from the surrounding atmosphere, which resulted in porosity of the weld metal (Figure 12). It was observed that the number of pores in the weld on the side of the root increases as the shielding-gas flow rate decreases. Figure 12 shows a cluster of pores in a weld made with the lowest shielding-gas flow rate.

No cracks were observed in the weld metal or HAZ. The microstructure of the base material (Figure 11b—area A) is polyhedral and fine-grained with slight heterogeneity, with grain sizes ranging from 50 to 78 µm. As the temperature increased, the grain size increased. The grain size ranged from 140 to 200 μm in the HAZ. In some parts of the HAZ, grains with acicular morphology were observed sporadically.

Based on metallographic tests, it was found that the following microstructural components occur in individual zones of the TIG-welded joint (Figure 11b):

- Weld—α′ phase (acicular structure) and α phase (granular structure);
- The HAZ—α phase and α′ phase (the grain size increases towards the weld axis);
- Fine grains of the α phase in the base metal.

Based on macro and microstructure tests, it can be concluded that joints made using the TIG method are characterised by the following:

- Larger grains of the α phase in the HAZ and in the weld;
- An acicular structure (α′ phase) in the HAZ;
- Much larger grain size in the acicular microstructure in the joint.

The volume percentage of porosity was calculated based on the cross-sections and was found to range from 1.18% to 3.46% for shielding-gas flows of 18 dm^3^/min and 4 dm^3^/min, respectively.

**Figure 13:**
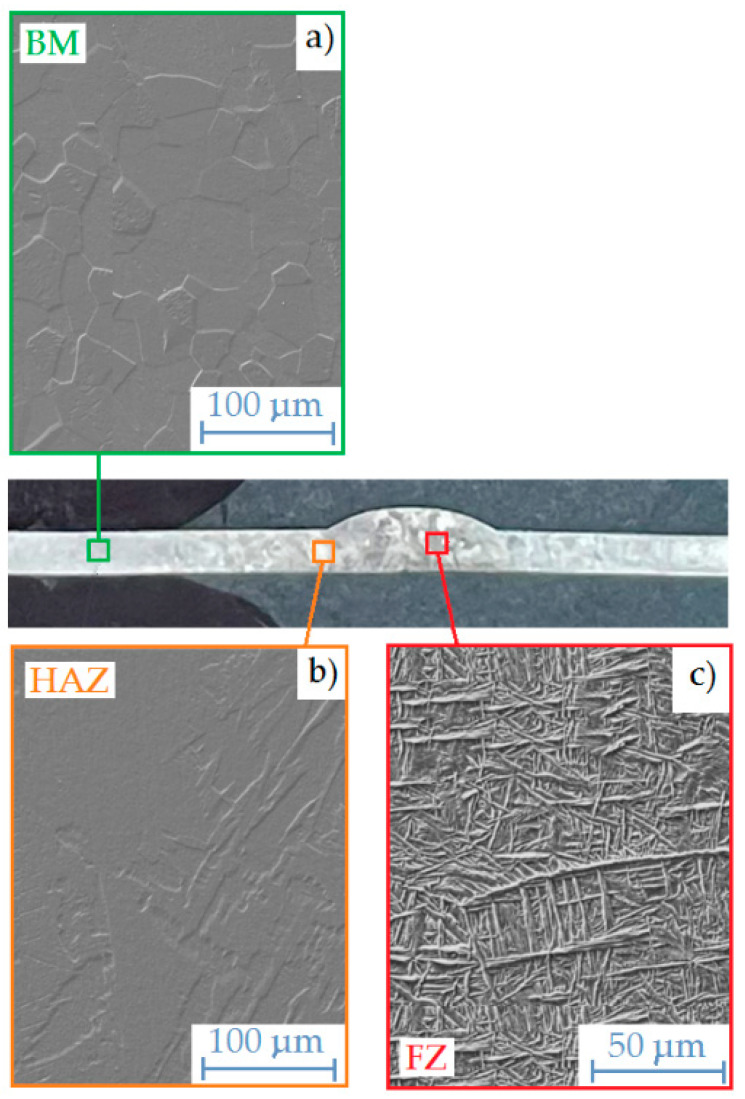
Microstructure of the TIG_1 weld: (**a**) base metal, (**b**) HAZ and (**c**) weld zone.

Thin needles and twins can also be observed in addition to columnar grains, which may be due to the presence of martensite in the weld material (Figure 13c).

The boundary of the fusion zone corresponds to the solidus temperature T_s_ (1668 °C for Grade 1). The microstructural changes occurring during the solidification of the molten material usually lead to a structure significantly different from the original one (before melting). The weld microstructure shown in Figure 13b was characterised by coarser grains than the base material (Figure 13a). Near the fusion zone, the grains are elongated in the direction of the heat flow. In the joint area, the acicular microstructure is dominant (Figure 13c).

The welds solidify from the edge of the fusion zone. The solid/liquid interface moves towards the centre of the weld. After solidification is completed, the local microstructure changes, which also depends on the cooling rate. Titanium crystalizes in various crystal structures: α (low-temperature HCP) and β (high-temperature BCC). The allotropic transformation temperature is approximately 882 °C. Thus, after cooling, titanium undergoes a high-temperature (β phase) allotropic transformation to a low-temperature α phase structure. In a titanium alloy, the β → α transition temperature changes due to the presence of iron, oxygen and other impurities. At low cooling rates, the grain growth process occurs. At a cooling rate faster than the critical cooling rate, martensitic transformation occurs. The martensite transformation is diffusion free. The martensitic transformation in pure titanium takes place above a critical cooling rate of approximately 3000 K/s.

However, it is known that the critical cooling rate of the martensitic reaction for CP titanium can differ significantly from that for pure titanium. The difference is mainly due to the presence of iron, which is a strong β-stabilizer. The morphology of microstructure occurring in the FZ exhibit thin needles (Figure 13c). It can be concluded that the cooling rate occurring during the welding process was sufficient for the diffusion-free martensitic transformation. It was observed in the case of welding at the two lowest values of shielding-gas flow rates (6 and 8 dm^3^/min).

### 3.6. Scanning Electron Microscopy

**Figure 14:**
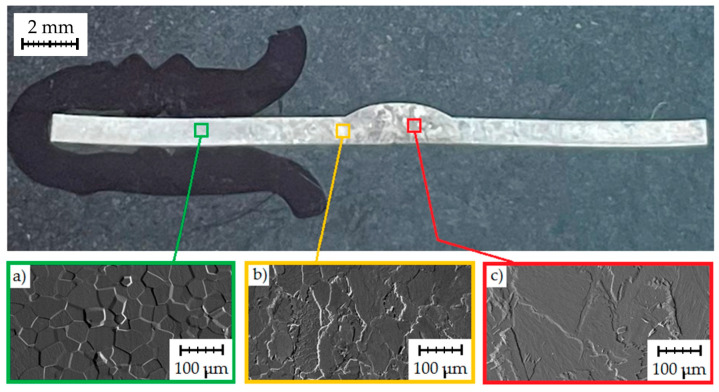
Microstructure of the TIG_3 welded joint: (**a**) microstructure of the base metal, (**b**) microstructure of the HAZ and (**c**) weld microstructure.

Based on scanning microscopy analysis, it was confirmed that the microstructure of the base material is characterised by grains that are polyhedral in nature, and the base material contains the α phase (i.e., a solid solution of alloying elements in α-titanium). Areas made of very fine polyhedral grains are clearly visible (Figure 14a). Figure 14c shows a view of the weld metal microstructure. The microstructure in this area lost its characteristic polyhedral character compared to the microstructure of the base metal. The grains gained a specific morphology through their growth, and a needle-like microstructure was clearly visible. Needle-like grains of the α phase were observed in the weld metal. Figure 14b shows the transition area between the weld metal and the base material. In the HAZ zone, there are large, coarse-grained polyhedral grains formed as a result of slow cooling.

### 3.7. XRD Analysis

**Figure 15:**
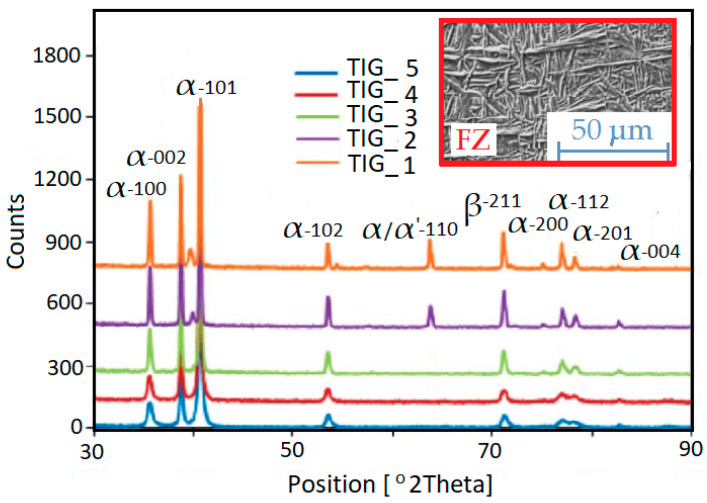
X-ray diffraction patterns across the welded joints.

The phenomenon of X-ray diffraction enables, among other things, the identification of the phase composition of crystalline substances, the analysis of crystallographic texture and the measurement of lattice parameters. Titanium undergoes an allotropic phase transition at 882 °C (alpha (HCP) ⇌ beta (BCC)) [20,21]. During welding, the material in the fusion zone is heated to a temperature above 882 °C, which causes the transition of the α phase into the β phase. As the weld crystallizes, the cooling rate from the β phase affects the resulting microstructure of pure titanium. Consequently, depending on the welding process parameters, the microstructure of titanium can vary significantly. Therefore, XRD analysis of the welded joints (Figure 15) was performed to determine which phases (either α-Ti or β-Ti) existed in the welds. Measurements were performed using a Cu Kα two-angle technique. The radiation reflected from the sample surface was recorded in the 2Ѳ range from 30 to 90° with a 2Ѳ measurement step of 0.1°. The scanning time was 10 s for one measurement step.

The main diffraction peaks reveal the presence of the α-Ti HCP phase. Alpha phases are identified for the HAZ and base metal. However, in the weld and at the boundaries of the fusion zone, the α-Ti BCC and β-Ti BCC phases are revealed. The α-Ti BCC and β -Ti BCC phases appeared when low shielding-gas flow rates were used in the tests. This occurred in the case of TIG_1 and TIG_2 specimens. This situation can be explained by an insufficient protection of the weld root against the influence of elements contained in the atmosphere. Titanium contains small amounts of iron and interstitial elements. Both non-metals (N, O, H, C) and metals (V, Cr, Al, Mo, Mn) can be dissolved in titanium, creating substitutional and interstitial solid solutions. The elements have different effects on the microstructure and properties of the resulting weld material. In CP titanium, martensite forms upon quenching from the high temperature BCC β phase field [43]. Martensite displays HCP α′ or orthorhombic α″ structure, depending on the composition [44].

## 4. Conclusions

Analysis of the influence of the amount of shielding-gas flow from the side of the root of the weld during TIG butt-welding of CP-Ti grade 1 titanium tubes was the topic of this article. The appearance of needle-like grains in the α′ phase in the weld metal was observed. It was observed that the amount of the α′ phase increased with reduction in the shielding-gas flow. Moreover, a coarse-grained multi-wall structure was found in the weld metal, larger than in the HAZ. The highest ultimate tensile strength (R_m_ = 481 MPa) was recorded when using a shielding-gas flow of 18 dm^3^/min. As expected, the lowest tensile strength was observed when the weld pool was not sufficiently shielded during welding (R_m_ = 420 MPa). The lowest microhardness in the weld of the welded joint (approximately 150–160 HV1) was recorded with increased gas shielding of the weld pool (12–18 dm^3^/min). For the lowest value of shielding-gas flow (6 dm^3^/min), there was a significant increase in the weld hardness (over 200 HV1).

The increase in the microhardness of the weld and the HAZ compared to the base metal is mainly related to the increase in the amount of acicular structure (α’ phase). On the contrary, the maximum porosity in the weld was observed for lower gas flow values. Better protection of the root of the weld during TIG welding (above 12 dm^3^/min) would avoid the occurrence of gas bubbles and increase the strength of the welded joint. Gas flow from the side of the root of the weld of 12 dm^3^/min is the optimal gas flow rate for TIG welding of Grade 1 pure titanium. Such gas flow from the side of the root of the weld protects the weld against oxidation.

The microstructure of the resulting joints of CP titanium tubes consists of small, equiaxed α grains. As a result of welding, martensite and a feathery α-titanium phase are formed in in the FZ. Both the amount of α phase with feathery morphology and martensite in the weld decreases with increasing shielding-gas flow rate. Phase analysis showed the presence of only α phase in the HAZ of the weld.

The research presented in this paper will be supplemented in the future with precise quantitative analysis (grain size) of the weld zone using morphology of the FZ by using simple geometric figures (patterns) whose shape can be defined by providing a small set of parameters: diameter, side length, height, width, etc. The pilot studies presented in this article confirmed that to ensure optimal tensile strength of TIG-welded joints, the shielding-gas flow rate should be different in both sides of the weld. Future studies will also focus on the synergistic effect of welding speed and shielding-gas flow rate on the hardness and tensile strength of the TIG-welded butt joints.

## Figures and Tables

**Table 1:** Chemical composition (% mas.) of CP (Grade 1) titanium.

C	Fe	N	H	O	Ti
0.024	0.096	-	-	-	Balance

**Table 2:** Selected mechanical properties of CP (Grade 1) titanium.

Yield Stress R_p0.2_, MPa	Ultimate Tensile Stress R_m_, MPa	Elongation A_50_, %	Hardness HV
345	445	18	132

**Table 3:** Welding parameters.

Sample Designation	Gas Flow from the Side of the Root of the Weld (dm^3^/min)
TIG_1	6
TIG_2	8
TIG_3	12
TIG_4	16
TIG_5	18

**Table 4:** Ultimate tensile stress of the welded joints.

Sample Denotation	R_m_, MPa	Elongation ΔL, mm
TIG_1	420 ± 7	33.9 ± 0.8
TIG_2	435 ± 5	35.3 ± 0.9
TIG_3	443 ± 10	36.4 ± 1.0
TIG_4	460 ± 8	37.5 ± 1.1
TIG_5	481 ± 6	39.0 ± 1.2

## Data Availability

No new data were created or analyzed in this study. Data sharing is not applicable to this article.
